# BC2L-C N-Terminal Lectin Domain Complexed with Histo Blood Group Oligosaccharides Provides New Structural Information

**DOI:** 10.3390/molecules25020248

**Published:** 2020-01-07

**Authors:** Rafael Bermeo, Anna Bernardi, Annabelle Varrot

**Affiliations:** 1 Univ. Grenoble Alpes, CNRS, CERMAV, 38000 Grenoble, France; rafael.bermeo@cermav.cnrs.fr; 2 Università degli Studi di Milano, Dip. Chimica, via Golgi 19, 20133 Milano, Italy; anna.bernardi@unimi.it

**Keywords:** TNF-like lectin, fucosides, blood group antigen, crystallography

## Abstract

Lectins mediate adhesion of pathogens to host tissues, filling in a key role in the first steps of infection. Belonging to the opportunistic pathogen *Burkholderia cenocepacia*, BC2L-C is a superlectin with dual carbohydrate specificity, believed to mediate cross-linking between bacteria and host cells. Its C-terminal domain binds to bacterial mannosides while its N-terminal domain (BCL2-CN) recognizes fucosylated human epitopes. BC2L-CN presents a tumor necrosis factor alpha (TNF-α) fold previously unseen in lectins with a novel fucose binding mode. We report, here, the production of a novel recombinant form of BC2L-CN (rBC2L-CN2), which allowed better protein stability and unprecedented co-crystallization with oligosaccharides. Isothermal calorimetry measurements showed no detrimental effect on ligand binding and data were obtained on the binding of Globo H hexasaccharide and l-galactose. Crystal structures of rBC2L-CN2 were solved in complex with two blood group antigens: H-type 1 and H-type 3 (Globo H) by X-ray crystallography. They provide new structural information on the binding site, of importance for the structural-based design of glycodrugs as new antimicrobials with antiadhesive properties.

## 1. Introduction

Nosocomial infections, also known as healthcare-associated infections (HCAIs), have always been part of the public health scene. They represent an alarming problem due to the rise of multidrug-resistant (MDR) pathogens. They are correlated with increased hospitalization, morbidity, mortality, and financial burden. *Burkholderia cenocepacia* is one of the notorious bacteria associated with this issue. This opportunistic pathogen is not only a widespread Gram-negative and biofilm-forming bacterium, but also a MDR bacterium with intrinsic resistance to multiple classes of antibiotics [1,2]. It represents a major hazard to hospitalized patients immunocompromised, affected with cystic fibrosis, or critically ill in the intensive care units [3,4]. *B. cenocepacia* belongs to the *Burkholderia cepacia* complex (BCC), a compendium of human pathogens from the *Burkholderia* genus that can potentially elicit the “cepacia syndrome” in patients, leading to a rapid decline of pulmonary function often leading to fatal outcome [5,6]. *B. cenocepacia* is one of the BCC species most frequently isolated from patients.

As other opportunistic bacteria, *B. cenocepacia* employs carbohydrate binding proteins (i.e., lectins) as virulence factors to target host tissues through recognition and subsequent adhesion to the glycoconjugates on the cell surface [7]. Among the different lectins produced by this bacterium, BC2L-C (272 amino acids) stands out to our interest. It was initially identified due to the strong homology of its C-terminal to LecB, a fucose binding lectin belonging to *Pseudomonas aeruginosa*, an even more notorious opportunistic bacterium involved in HCAIs [8]. BC2L-C also presented at its N-terminus a domain of unknown function, which was later revealed to be also a lectin domain (BC2L-CN). Establishing a new lectin family, BC2L-CN showed specificity for fucosides, especially blood group antigens, for which the measured affinity reached micromolar range. Blood group antigens are commonly targeted by lectins during microbial infections since they are highly present on the surface of epithelial cells, mucosa, and mucus [9]. BC2L-CN was the first lectin to display a trimeric arrangement of the jelly roll fold primarily observed in tumor necrosis factor α (TNF-α). A novel binding site and binding mode for fucosides was also uncovered at each interface between two protomers, which implicated an essential arginine residue [10]. Furthermore, BC2L-C was the first superlectin to be characterized with its two lectin domains assembling to form a final hexameric structure [11].

Lectins play a key role in microbial adhesion to host cells and between bacterial cells during biofilm formation, hence, antiadhesive glycodrugs or glycomimetics inhibiting lectins are attracting increasing attention as novel antimicrobials [12,13]. In this context, and due to its affinity for human oligosaccharides, BC2L-CN becomes a relevant target. Nevertheless, prior to any probing, additional information is required on its interactions with oligosaccharides. The original construct to produce BC2L-CN in recombinant form allowed crystallization only with the α-methyl fucoside. It consisted of the first 156 residues of BC2L-C with an additional C-terminal tag of 31 amino acids [10]. Structural analysis revealed that only the first 130 residues constitute the N-terminal domain of BC2L-C. The remaining residues belong to the linker between the two lectin domains and tag. These residues could not be observed in the crystal structure, hinting at high flexibility, which would explain the relative instability of the purified protein. Likewise, the impossibility to crystallize it with oligosaccharides could be attributed to this structural shortcoming, as those residues could hinder oligosaccharide binding. In order to overcome these limitations and expand the structural study of the protein, a shorter construct was designed comprising only the first 132 residues of BC2L-C and a cleavable N-terminal fusion to help expression and purification.

Here, we present the cloning, expression, and purification of the new construct of BC2L-CN (rBC2L-CN2) as well as the evaluation of its binding affinity for different fucosides by isothermal microcalorimetry (ITC). We also describe its structures in complex with H-type 1 and Globo H blood group antigens at 1.6 and 1.9 Å resolution, respectively, with details on the new interactions observed.

## 2. Results

### 2.1. New Construct for Recombinant of BC2L-CN

**Figure 1:**
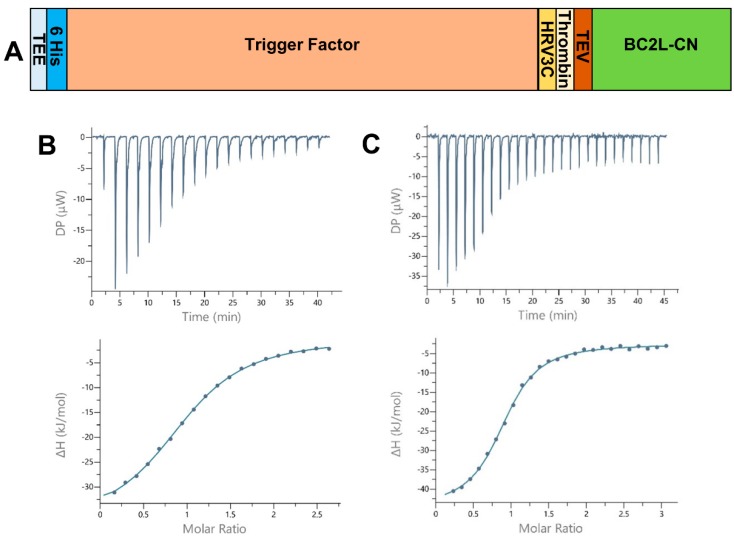
(**A**) Schematic of the expression construct of BC2L-CN2 in pCold-TEV. The N-terminal fusion presents a translation enhancing element (TEE), the trigger factor (TF) and 3 protease sites (human rhinovirus 3c (HRV 3c), thrombin, and tobacco etch virus (TEV)). The full construct leads to a protein of 605 and 134 amino acids before and after cleavage with the TEV protease. (**B**,**C**) Isothermal microcalorimetry data. Titration of rBC2L-CN2 (314–350 µM) by H-type 1 tetrasaccharide ((**B**), 4 mM) and Globo H hexasaccharide ((**C**), 10 mM) at 25 °C. The isothermal titration microcalorimetry (ITC) thermogram is displayed in the upper panel and the integration of data with curve fitted for “one binding site” model in the lower panel.

To improve upon the shortcomings of the previous construct of BC2L-CN on stability, flexibility, and difficulty to obtain crystal complexes with oligosaccharides, a new design was conceived. Based on the structural examination of first BC2L-CN structure (PDB 2WQ4), a construct featuring solely the ordered amino acids of the N-terminal domain was designed (1 to 131). The desired DNA sequence was amplified by PCR using the old construct as a template, purified, and subcloned into two vectors of expression: pET-TEV (Pet28a derivative) and pCold-TEV, using the restriction sites NdeI and XhoI. The pCold-TEV vector is a modified pCold-TF vector in which the factor Xa cleavage site (TATCGAAGGTAGG) was replaced with a TEV cleavage site (GAAAACCTGTATTTTCAGGGC) by mutagenesis using appropriate oligonucleotides [14,15,16]. The protein expressed by those vectors presented a N-terminal fusion that could be removed by proteolysis using the tobacco etch virus (TEV) protease and also included a 6-Histidin tag to ease purification. Successful expression was obtained overnight at 16 °C after induction with isopropyl-β-D-thiogalactoside (IPTG). Soluble protein was purified using immobilized nickel affinity chromatography thanks to the presence of the His-tag, over two steps: before and after TEV cleavage. While the TEV protease failed to cleave the 20 amino acids-long tag issued from pET-TEV, the larger fusion expressed using pCold-TEV allowed a conformation compatible with TEV cleavage, thus justifying its choice for further studies. It provided a 605 residues-long protein (66.4 kDa) with a N-terminal fusion (52.4 kDa) containing the trigger factor, a prokaryotic ribosome-associated chaperone (46 kDa), which aids co-translational folding of newly expressed polypeptides (Figure 1A). Size exclusion chromatography (SEC) was performed as a final purification step, leading to a 95% pure protein with an average yield of 5.2 mg.L^−1^ of culture medium. In this form, concentrated protein (up to 15 mg.mL^−1^–1.070 mM) was stable for several months both at 4 °C and −20 °C. Very little precipitation could be observed after one year storage in the fridge.

### 2.2. Physical Analysis of the rBC2L-CN2

Size exclusion chromatography on a methacrylate-based matrix (Enrich70, Bio-Rad, Marnes-la-Coquette, France) was used to remove the last contaminants, remaining uncleaved protein, and aggregates. It also allowed us to confirm the oligomerization and homogeneity of the protein. The molecular size of the sharp eluted peak corresponding to rBC2L-CN2 on SDS PAGE was approximated to 33 kDa by the means of a calibration curve (data not shown). This value was compatible with the expected trimer, which has an estimated molecular weight of 42 kDA. The weight discrepancy could be due to some nonspecific interactions of the lectin with the gel filtration matrix delaying the protein elution. This was observed before for other lectins when confronted to a saccharidic matrix [17].

To further confirm the trimeric arrangement, dynamic light scattering (DLS) measurements were performed at 22 °C on the purified protein. A monodisperse peak was obtained leading to a hydrodynamic radius of 2.94 nm which corresponded to a protein with a molecular weight of 41.9 kDa, matching the expected weight of the trimer (data not shown). The deletion of the 56 amino acids of the original construct did not have an impact on protein folding or on trimer formation of the N-terminal domain.

### 2.3. Affinity Analysis and Activity Validation

Isothermal titration calorimetry (ITC) measurements were then performed on rBC2L-CN2 to verify that its binding to carbohydrate was not modified. The affinity (dissociation constant) and thermodynamic contributions were obtained for the interactions with various human blood group antigens including H-type 1 tetrasaccharide (Fucα1-2Galβ1-3GlcNAcβ1-3Gal), Lewis Y pentasaccharide (Fucα1-2Galβ1-4(Fucα1-3)GlcNAc β1-3Gal), and Globo H hexasaccharide (Fucα1-2Galβ1-3GalNAcβ1-3Galα1-4Galβ1-4Glc), which presents H-type 3 antigen. Due to its similarity to l-fucose, the monosaccharide l-galactose was also analyzed. This experiment was suggested by a close analysis of the Fuc methyl group binding region in the available X-ray structures. Indeed, the fucose methyl group moiety was found in lipophilic contact with the side chain of Tyr 48, and it was flanked by the hydroxyl group of Thr83, whose side chain lined the pocket on the opposite side. This suggested that a hydroxyl group at Fuc C6 (leading to the L-Gal structure) may actually be accepted by the lectin. Some of the ITC titration values measured were compared to those previously obtained with the original construct, also reported in Table 1.

All thermograms started with strongly exothermic peaks, characteristic of enthalpy-driven interactions, followed by peaks decreasing in height while saturation was achieved (Figure 1B,C). When strong binding affinity led to a sigmoidal curve, the stoichiometry of binding was determined with values close to 1.0, indicating one sugar binding site per monomer. For low-affinity ligands, such as the l-galactose monosaccharide, the stoichiometry was fixed to 1.0. We started by testing Lewis Y and H-type 1, which previously presented the best affinities for BC2L-CN. The values obtained after a single measurement were within the expected range, proving that rBC2L-CN2 was functional (Table 1 and Figure 1B). Despite a lack of reliable thermodynamic data, the affinity for l-galactose could be estimated in the millimolar range, with a K_d_ of 2 mM, which was similar to what was previously measured for alpha-methyl-l-fucoside (K_d_ = 2.7 mM) [10]. This supported our structure-based hypothesis that a hydroxyl substitution at fucose C6 would not be detrimental for binding and offers a useful starting point for the design of unnatural ligands of BC2L-C N-terminal domain. We also determined the thermodynamic parameters for the interaction of rBC2L-CN2 with Globo H. This hexasaccharide contains the H-type 3 antigen (Fucα1-2Galβ1-3GalNAc), previously shown to be recognized by BC2L-CN in cell binding assays and in glycan arrays (Figure 1C) [10,18]. We measured a K_d_ of 26 µM, which classified Globo H as the best ligand to date for BC2L-CN. The thermodynamic contributions indicated an enthalpy-driven increase of affinity for the larger ligands, but also strong entropic penalties associated with Globo H ligand.

### 2.4. Resolution of rBC2L-CN2 Structure in Complex with Oligosaccharide

In order to better understand the specificity of BC2L-CN towards the motif Fucα1-2Galβ1-3 and to obtain its atomic basis, we co-crystallized rBC2L-CN2 with H-type 1 tetrasaccharide and Globo H hexasaccharide. Clusters of plate crystals were obtained in a few days using a concentrated solution of sodium citrate. Single plates were cryoprotected using 2.5 M sodium malonate pH 5 and diffracted to high resolution. Both structures were solved by molecular replacement. The structure of the H-type 1 complex belongs to space group R32:h (H32, a = b = 42.7 Å, c = 308.6 Å) with one monomer in the asymmetric unit, whereas the one of the H-type 3 complex belongs to the C2 space group (a = 74.4 Å, b = 42.9 Å, c = 102.6 Å and β = 96.0°) with one trimer in the asymmetric unit. Statistics on data and refinement are summarized in Table 2. No crystal has been obtained to date with Lewis Y as a ligand.

#### 2.4.1. Overall Structure

**Figure 2:**
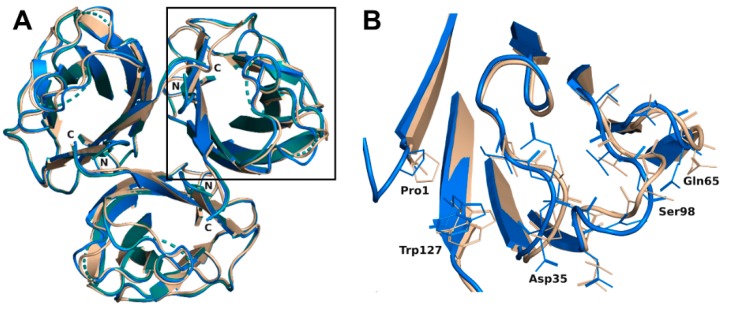
(**A**) Overlay of the overall trimer of rBC2L-CN2 in the complex with H-type 1 (green, 6TID, generated by crystal symmetry) or Globo H (blue, 6TIG) with the original trimer (beige, 2WQ4). (**B**) Zoom on the loop conformation changes observed in the protein chain B in the complex with Globo H.

The trimer formed in each complex (crystal symmetry were applied to build the trimer for the H-type 1 complex) was almost equivalent with a root mean square deviation (rmsd) of 0.16 Å for 393 aligned residues and was very similar to the trimer of the previous construct with an rsmd of 0.48 Å for 379 aligned residues [19]. Only minor differences were noticed on the bottom face of the jelly roll presenting the N- and C-termini of rBC2L-CN2. Both ends interacted through hydrogen bonds. Those interactions would not exist in the native BC2L-CN, since they involved residues from the added TEV cleavage site that remained after cleavage of the protein fusion. Nevertheless, they appeared to stabilize the domain. The small subsequent changes in the conformation of the termini, in particular for Trp127, impacted the nearby surface loops (Val28-Gly35, Asn64-Gln65, and Val96-Thr101) and resulted in small rigid body movements from 0.6 to 1.2 Å. Flipping of Gly97 was also observed, leading to alternative orientations of Ser98. In the H-type 1 complexed structure, the electron density for Ala31, Gly32, and Ser98 was too poor to allow modeling with suitable geometry. So, those residues were omitted in the final coordinates (Figure 2). No changes relative to the original construct were observed on the top face of the jelly roll where the binding site was located.

#### 2.4.2. Oligosaccharide Binding Interactions

**Figure 3:**
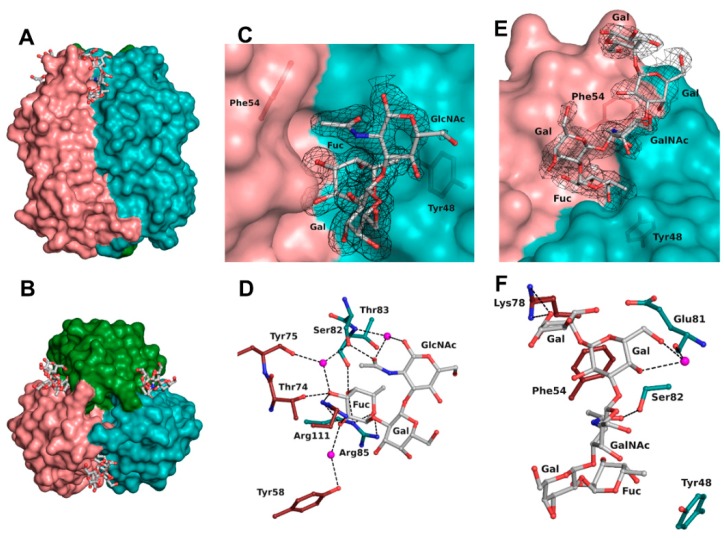
BC2L-CN2 binding site. Surface representation of the trimer of BC2L-CN2 in complex with Globo H, view from the side (**A**) and from the top (**B**). Surface representation of the binding interface between protomers B and C with the electron density displayed around H-type 1 (**C**) and Globo H (**E**) antigens at 1 σ (0.43 and 0.37 eA^3^, respectively. Zoom on the interactions of BC2L-CN2 with H-type 1 (**D**) and Globo H (**F**) antigens. Protein residues are colored by protomer chain. Waters are represented as spheres.

Examination of the first electron density revealed without ambiguity binding of the oligosaccharides. Regarding the structure in complex with H-type 1 tetrasaccharide, only three of the four sugar units could be correctly modelled. The reducing galactose was too disordered as a result of its exposition to the solvent and the quality of the residual electron density was insufficient for modelling. For the structure in complex with Globo H hexasaccharide, the four units were modelled in every protein chain and the fifth one only in protein chains B and C. No electron density was visible for the reducing glucose, which, likewise, was completely solvent exposed (Figure 3).

The binding site was found at the interfaces between two protomers and was located on the top side of the jelly roll β-sandwich in a shallow groove (Figure 3A,B). The bottom of the binding site was principally made from residues from a surface loop (Glu81-Arg85) and also Tyr48. The orientation of the oligosaccharide was imposed by a wall made from Asn53, Phe54, Tyr58, Thr74, Lys78, Val110, and Arg111 from the neighboring protein chain. The fucose residue was buried in the protein, whereas the other sugar units of the oligosaccharide were pressed against the protein wall on one side while the other one was completely exposed to the solvent. As previously observed, the fucose was involved in extensive H-bonding with the protein and accounted for the majority of the interactions (Table 3 and Figure 3D). All oxygen atoms established direct or water-mediated contacts with the side chain of Arg85 (O4 and O5) and Ser82 (O3 through a water molecule) and the main chain of Thr83 (O4) from one protomer, as well as with the side chain of Arg111 (O2 and O3), Thr74 (O3), Tyr58 (O2 through a water molecule), and the main chain of Tyr75 (O3 through a water molecule) from the neighboring protomer involved in the binding site interface. Additionally, a hydrophobic interaction between the C6 methyl and the aromatic ring of Tyr48 as well as the two waters molecules that mediate hydrogen bonding were observed, matching what was observed in the previous crystal structure (2WQ4) [10].

The next sugar residue, an α1-2 linked galactose (Gal2), was very solvent-exposed and did not interact with the protein. The following N-acetylated sugar, GlcNAc in H-type 1 or GalNAc in H-type 3, interacted mainly with its acetyl group. The acetyl oxygen was hydrogen-bonded to the side chain of Ser82 while the methyl group was engaged in a hydrophobic interaction with the aromatic ring of Phe54 from the neighboring protein chain. In some chains, the acetyl group’s oxygen also established a hydrogen bond mediated by a water molecule with both the next glycosidic oxygen and the main chain of Thr83. For the remaining sugar units in the Globo H structure, most of the interactions were seen in protein chain C and are depicted in Figure 3F. The O6 and O4 hydroxyls of the fourth sugar (Gal4) made a water-mediated hydrogen bond with the main chain oxygen of Glu81. In the α1-4 linked galactose (Gal5), only one interaction was observed between the O6 and the side chain of Lys78 from the neighboring chain. Those interactions with Gal4 and Gal5 were not seen in the protein chain B as a result of a different orientation of the O6 of Gal4 and of the terminal nitrogen of Lys78. 

All sugar units presented the expected chair conformation and a good fit in the electron density as checked in Privateer [20]. The dihedral angles of glycosidic linkages were compatible with low-energy conformation, apart from the Galα1-4Gal connection that was in the vicinity of the second main low-energy minimum (Table 4) [21]. The Fucα1-2Gal linkage adopted dihedral angles consistent with the primary low-energy conformation, while for the other linkage, they corresponded to those of the secondary low-energy minimum. 

**Figure 4:**
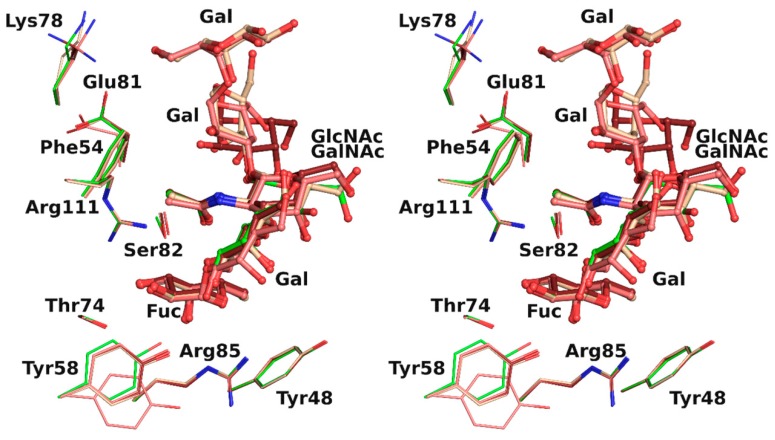
Overlay in wall eye stereo of each binding site observed in rBC2L-CN2 structure with H-type 1 (green) or Globo H (H-type 3) with carbon atoms colored in dark red, beige, and pink for chain A, B, and C, respectively.

The overlay of all the binding sites shows that the conformation obtained for the H-type 1 trisaccharide was the one mainly observed in the H-type 3 trisaccharide (Figure 4). In protein chain C, there was a slight rotation around the C1-O2 bond of the first glycosidic linkage by ~22°, which led to a rigid body shift of the subsequent saccharide units Gal2, GalNAc3, and Gal4 by ~0.9 Å. This did not, however, impact strongly on ligand binding since both the fucose and the N-acetyl of GlcNAc or GalNAc that were involved in the major conserved interactions presented equivalent position. Only the aforementioned water-mediated interaction between the oxygen of the acetyl group and the glycosidic oxygen was lost. The GalNAc hydroxymethyl group had a different orientation in each protein chain and only one was involved in the water-mediated hydrogen bonds described above. The fifth sugar unit, Gal5, was well superposed in the two chains where it was observed, despite the rigid body shift observed for the other residues. In protein chain A no density was visible after Gal4, which presented different torsion angles than in the other chains, resulting in a more solvent-exposed sugar (Table 4).

## 3. Discussion

We designed a novel construct for BC2L-CN to allow crystallographic studies with oligosaccharides and to improve stability of the recombinant domain. Another construct encompassing only BC2L-CN residues created by Tateno et al. doesn’t provide details on its length or the final protein yield [22]. Consequently, we chose to add a cleavable N-terminal fusion to help protein expression and allow purification by affinity on a nonsugar-based matrix. The rBC2L-CN2 met our expectations in terms of stability, as it is now stable over a year at 4 °C. The extra 50 amino acids of the original construct seem, therefore, to be indeed responsible for the precipitation formerly observed after merely two weeks of storage. The trimer was formed correctly and the overall structure was marginally influenced by the interactions between the new N- and C-termini. Conversely, these termini locally influenced the conformation of neighboring surface loops, but this did not reach or alter the binding site functionality, as confirmed by ITC measurements. The latest revealed a correlation between gain in affinity and oligosaccharide length that is not uncommon for bacterial lectins but remains to be fully clarified [23]. As is usually observed for lectin–carbohydrate pairs, the interaction was driven by enthalpy, with unfavorable entropy. The enthalpic gain can be linked to the novel interactions established by the additional carbohydrate moieties, while the entropic gain observed could be linked to the loss of flexibility upon binding or to desolvation effects. Nevertheless, we showed that BC2L-CN had the strongest affinity for Globo H, which was listed as one of the best ligands of BC2L-CN on glycan arrays, along with H-type 1 and Lewis Y [10]. 

Using rBC2L-CN2, we were able to obtain molecular information on the binding of two relevant oligosaccharides, to better characterize the protein carbohydrate binding site, where to date only the fucose subsite has been depicted [10]. The structures of rBC2L-CN2 in complex with H-type 1 and Globo H allowed us to identify a network of interactions composed of hydrogen bonds and hydrophobic contacts also implicating structural waters molecules, often involved themselves as mediators of protein–ligand interactions (Table 3). As observed in Figure 4, the full binding site was quite extended and solvent exposed and accommodated up to five carbohydrate units. The additional sugar moieties had little interaction with the protein surface but still allowed the identification of novel protein determinants and water molecules to be taken into consideration for ligand design, such as Phe54, Lys78, and Glu81. It is striking to notice that with only one strong hydrogen bond and one hydrophobic contact (belonging to the N-acetyl group of GlcNAc/GalNAc), the affinity was improved by two orders of magnitude, from 2.7 mM for l-fucose to 55–77 µM for the tetrasaccharide. Equally striking is to note that no interactions were made by Gal2 and that the ones observed for Gal4 were not conserved: Rather than interacting with the residues of the binding site, these additional sugars seemed to play the role of a frame, allowing a handful of essential interactions to fall into place. 

Both the H-type 1 and Globo H antigens are present on pluripotent cells and can be used as glyco-markers. The Globo H glycosphingolipid is also highly expressed on malignant tissues, particularly in breast and small cell lung cancers [24,25]. Globo H presents the H-type 3 antigen (Fucα1-2Galβ1-3GalNAc) which only differs from H-type 1 antigen (Fucα1-2Galβ 1-3GlcNAc) by the epimerization of GlcNAc at C4 position. Since no interaction implying the O4 hydroxyl is observed, the recognition of both antigens is equivalent at the structural level. Accordingly, BC2L-CN is an excellent probe for detecting or labelling undifferentiated human pluripotent stem cells or human embryonic stem cells [18,22,26]. Thus, the newly created BC2L-CN2 has great potential as a molecular tool for the detection of the Fucα1-2Galβ 1-3GlcNAc/GalNAc motif.

Unfortunately, no crystal complex could be obtained with the Lewis Y antigen (Fucα1-2Galβ1-4(Fucα1-3)GlcNAc), another high-affinity ligand of BC2L-CN that contains two terminal fucose moieties. Since H-type 2 was not well recognized by the protein, it is most probable that BC2L-CN bound preferentially the Fucα1-3 and not the Fucα1-2 presented by Lewis Y [10]. More insights on the binding mode of Lewis Y will come with further experimentation: Measurements of the affinity with the disaccharide Fucα1-3GlcNAc and continued crystallization trials.

The information gathered here can be used for the design of fucose-based glycodrugs towards BC2L-CN, since elimination of drug-resistant pathogens, such as *B. cenocepacia*, is still a critical issue nowadays, as available medication is limited. Numerous pathogens, in particular bacteria, exploit interactions between host-associated glycans and lectins for cell invasion and infection persistence by mediating adhesion to the host cells, and for pathogenesis by defining cell and tissue tropism [7,27]. Blocking the attachment of the pathogen to the host using lectin antagonists is a current route explored for the development of novel antimicrobial molecules (reviewed in [12,28,29]). Lectin inhibitors can also be used as antibiofilm molecules or for biofilm imaging [30,31]. Some lectins play indeed an important role in the formation and the maintenance of biofilms, a hallmark of chronic infections and resistance against antimicrobials (antibiotics or antifungals). 

Following this theme, the structural information related to the spatial arrangement of the different binding sites (referred as lectin topology) can now facilitate the design of selective structure-based multivalent inhibitors. Multivalency, a common lectin feature, usually counterbalances the weak affinities observed in carbohydrate–lectin interactions, such as the values we have obtained for monosaccharides. Mediating several weak binding events simultaneously usually results in strong avidity and, therefore, increased affinity. Based on our structural data on BC2L-CN, a multivalent compound should present a fucose mimic to insure specificity; furthermore, the addition of an aglycone binding to the nonfucose subsites, in particular the one occupied by Gal5, could improve selectivity and affinity. Branching could be also attempted on the other side of the fucose binding site. As BC2L-CN is trivalent with the fucose binding sites 25 Å apart and at 11 Å from the top face of the trimer, a linker of appropriate length between the fucoside moieties and a scaffold for multivalency can now be rationally chosen.

As a result of its dual specificity and structural features, the superlectin BC2L-C from *B. cenocepacia* is indeed an interesting target for the design of antiadhesive or antibiofilm inhibitors. The dual carbohydrate specificity of this hexameric protein hints at its involvement in cross-linking. It presents several opposing binding surfaces: The N-terminal domain trimers (two, facing top and bottom of the hexamer) are selective for fucosides; conversely, the C-terminal domain dimers (three, forming a central belt) specifically bind to mannosides [11]. BC2L-C C-terminal domain would allow binding of the lectin to the bacterial cell wall through recognition of manno-configured carbohydrates, as observed for its homolog BC2L-A, another soluble lectin from *B. cenocepacia* [32]. On the other hand, the N-terminal domain would target fucosylated ligands on host cells, in particular human blood group oligosaccharides. BC2L-A and especially LecB have been targets for the design of both mono- and multivalent antiadhesive compounds for over a decade. They are derived either from fucose or mannose (reviewed recently in [12]) and could be effective on BC2L-C. Nevertheless, to date only C-fucosides-calix[4]arene (1-3-alternate) have been tested on BC2L-C [33]. This inhibitor is able to crosslink *B. cenocepacia* cells and inhibits BC2L-C-induced hemagglutination. As the compound does not inhibit hemagglutination induced by LecB, it most probably binds to BC2L-CN, suggesting also a role of this domain into cell cross-linking and, hence, in biofilm formation. Since both BC2L-C domains have millimolar affinity for l-fucose (personal communication of O. Sulak), more studies seem necessary to understand the mode of binding of inhibitors and to investigate the role of both domains in the biofilm of *B. cenocepacia*.

Key molecular determinants involved in the binding of BC2L-CN to human oligosaccharides are now identified. Further probing will permit us to complete our understanding of BC2L-CN’s binding to human epitopes and, in particular, to Lewis Y. The data gathered here are being used for the conception of glycodrugs specific to BC2L-CN, which will then be synthetized, and their potential evaluated as antiadhesives or antibiofilm agents.

## 4. Materials and Methods

### 4.1. Protein Expression and Purification

The DNA sequence encoding for BC2L-C-Nter comprising amino acids 2 to 132 was amplified by PCR with purposely designed primers using previous construct as template and 5′- CTTCATATGCCGCTGCTGAGCGCCAGTATCG-3′ and 5′-TACTCGAGTTATGCCGCGGTGCC CCAAATCG-3′ as forward and reverse primers, respectively (restriction sites are underlined) [10]. The PCR product (ca. 400 base pairs) was purified from 1% agarose gel using Nucleospin Gel and PCR Clean-up kit (Macherey-Nagel, Hoerdt, France) using manufacturer instructions. The gene product and homelab vectors of interest (pET-TEV [14] and pCold-TEV [16]) were digested with NcoI and XhoI restriction enzymes (New England Biolabs, Evry, France) for 1 h at 37 °C, purified, and ligated at room temperature using the Takara mix. The pCold-TEV originates from the pCold-TF vector (Takara Bio Europe, Saint Germain en Laye, France) where the enterokinase site was replaced by tobacco etch virus (TEV) cleavage site by PCR using the 5′-cgcggtagtggtggtgaaaacctgtattttcagggccatatggagctcggtacc-3′ and 5′-accaccactaccgcgtggcaccagacccgc-3′ as forward and reverse primers, respectively, with the PrimeSTAR Max DNA polymerase (Takara Bio Europe, Saint Germain en Laye, France) according to manufacturer instructions.

After transformation by heat shock, *Escherichia coli* BL21 Star (DE3) cells harboring the plasmid were cultured in Luria Broth (LB) broth medium supplemented with 100 µg/mL ampicillin at 37 °C under constant shaking. At OD_600nm_ = 0.4, the incubator temperature was decreased to 16 °C and when OD_600nm_ reached 0.7, the protein expression was induced overnight by the addition of 0.1 mM IPTG. Then, cells were centrifuged at room temperature, 5 min at 5000× *g*, and the resulting pellet was weighed. Each g of wet cell pellet was resuspended with 5 mL of Buffer 1 (Tris-HCl 50 mM, NaCl 100 mM, pH 8.5) prior to treatment with DENARASE^®^ endonuclease (c-LEcta GMBH, Leipzig, Germany) for 10 min at room temperature on a rotating wheel. The cells were lysed by pressure at 1.9 MPa using a one-shot table-top cell disruptor (Constant Systems Ltd.). The lysate was centrifuged 30 min, 24,000× *g* at 4 °C, and the resulting supernatant filtered through a 0.45 µm polyethersulfone (PES) syringe filter prior to loading on a 5 mL HisTrap™ fast flow (FF) column (GE Heathcare Life Sciences, Marlborough, MA, USA) equilibrated with buffer 1 for affinity chromatography using NGC system (Bio-Rad, Marnes-la-Coquette, France)). After washing the unbound proteins with buffer 1, rBC2L-CN2 was eluted using a 20 column volumes (CV) gradient of 0–500 mM imidazole. Fractions containing the protein were pooled after examination on 15% SDS-PAGE gel. The imidazole was removed using a PD10 desalting column (GE Healthcare Life Sciences, Marlborough, MA, USA). The protein was concentrated by centrifugation (Vivaspin 3KDa, Sartorius, Goettingen, Germany) to at least 0.7 mg.mL^−1^ before addition of TEV protease (1:50 *w*/*w*, enzyme:protein ratio), 1 mM ethylenediaminetetraacetic acid (EDTA) and 0.5 mM tris(2-carboxyethyl)phosphine (TCEP) for tag cleavage overnight at 19 °C [15]. The sample was again submitted to affinity chromatography (same conditions as previously) to separate two fragments of 14 kDa and 52 kDa corresponding to the target protein and its cleave fusion, respectively (assessed by SDS–PAGE). After concentration by centrifugation as previously described, the protein concentration was determined by UV absorbance at 280 nm with a NanoDrop 2000 spectrophotometer (Thermo Scientific, Illkirch-Graffenstaden, France).

SEC was performed on an ENrichTM SEC 70 10 × 300 column (Bio-Rad, Marnes-la-Coquette, France)) using a NGC™ systems (Bio-Rad Ltd.). The analytical column was pre-equilibrated with 20 mM Tris-HCl pH 7.0 and 100 mM NaCl, optimized for protein stability via thermal shift assay (TSA). The volume for the sample injections was 240 µL and the flow rate was 1.0 mL/min. A column calibration curve using gel-filtration standards (GE Healthcare, Life Sciences) was performed to allow the calculation of the protein molecular weight.

### 4.2. ITC Measurements

All experiments were performed at 25 °C with an ITC200 isothermal titration calorimeter (Microcal-Malvern Panalytical, Orsay, France). The rBC2L-CN2 and sugars were dissolved in the same buffer composed of 100 mM Tris HCl pH 7.0 and 100 mM NaCl. A total of 20 to 38 injections of 1 µL of sugar solution (10 or 15 mM) were added at intervals of 100 or 200 s while stirring at 850 rev/min^−1^ in the 200 µL sample cell containing the protein, at 250 or 340 µM. The experimental data were fitted to a theoretical titration curve using the supplied software Origin 7. They permitted us to determine in one experiment affinity (i.e., association constant, Ka), binding enthalpy (∆H), and stoichiometry (n). Free energy change (ΔG) and entropy contributions (TΔS) were derived from the equation ΔG = ΔH − TΔS = −RT ln Ka (with T the absolute temperature and R = 8.314 J mol^−1^ K^−1^. For experiments in ligand excess, the stoichiometry was fixed to 1. Two experiments were performed for l-galactose, three for Globo H hexasaccharide (H-type 3), and only one for H-type 1 tetrasaccharide or Lewis Y pentasaccharide. The oligosaccharides were purchased from Elicityl, Crolles, France.

### 4.3. Crystallization, Data Collection, and Structure Determination

Oligosaccharides (H-type 1 tetrasaccharide and Globo H hexasaccharide) at 10 mM in water were added to rBC2L-CN2 at a concentration of 5 mg.mL^−1^ such that the final ligand concentration was 1 mM. After incubation at room temperature (22 °C) for at least 1h, crystallization conditions were screened using the vapor diffusion method and 2-μL hanging drops containing a 50:50 (*v*/*v*) mix of protein and reservoir solution. The screens used included: BCS Eco Screen, Eco Structure Screen 2, Morpheus I-carboxylic acids, and MIDAS (Molecular Dimensions Ltd., Sheffield, UK). Crystals were obtained in a few days from solution 48 of the Structure Screen 2 and optimized using 1.2–1.4 M sodium citrate pH 7.0. Both complexes led to clusters of plates which were broken, transferred to 2.5 M sodium malonate (CryoProtX, Molecular Dimensions Ltd.) for cryoprotection, and flash-cooled in liquid nitrogen prior to data collection. H-type 1 complexed data were collected at European Synchrotron Radiation Facility (ESRF), Grenoble France, on beamline FIP-BM30A using a ADSC Q315r detector (Area Detector Systems Corporation, Poway, CA, USA),while those for the Globo H complex were collected on the beamline Proxima 1, synchrotron SOLEIL, Saint Aubin, France, using an Eiger 16 m detector (Dectris, Baden, Switzerland).

The data were processed using XDS and XDSME [34,35]. All further computing was performed using the CCP4 suite [36]. The coordinates of the monomer of PDB-ID 2WQ4 were used as a search model to solve both complexed structures of rBC2L-CN by molecular replacement using PHASER [37]. Refinement was performed using restrained maximum likelihood refinement and REFMAC 5.8 [38] iterated with manual rebuilding in Coot [39]. Five percent of the observations were set aside for cross-validation analysis with the same set used for both structures. Hydrogen atoms were added in their riding positions and used for geometry and structure-factor calculations. The final model was validated with the wwPDB Validation server, https://validate-rcsb-1.wwpdb.org/, and the sugar conformation was checked in Privateer [20]. The coordinates were deposited in the Protein Data Bank (PDB) under codes 6TID and 6TIG for H-type 1 and Globo H complex structures, respectively. Data and refinement quality statistics are summarized in Table 1.

## Figures and Tables

**Table 1:** Affinity measurements for the binding of various carbohydrates to rBC2L-CN2 by isothermal microcalorimetry at 25 °C.

Ligand	n	K_d_ (μM)	−ΔG (kJ/mol)	−ΔH (kJ/mol)	−TΔS (kJ/mol)	Ref
Lewis Y	0.99 ^a^	52.6	24.4	43.3	18.8	This study
	0.98 ± 0.03	53.9 ± 2.9	24.4 ± 0.2	34.9 ± 0.3	10.5	[10]
H-type 1	1.01 ^a^	56.6	24.3	37.5	13.2	This study
	0.93 ± 0.02	77.2 ± 1.5	23.5 ± 0.2	23.0 ± 0.3	−0.5	[10]
l-galactose	1 ^b^	2000	NA	NA	NA	This study
GloboH (H-type 3)	0.83 ± 0.06	26.05± 1.7	26.1 ± 0.2	46.1 ± 3.9	20.1	This study

NA: not applicable. Averages values and experimental errors are given when at least two independent measurements were made. ^a^ In the case of H-type 1 and Lewis Y, parameters result from unique measurements only, as they were in the same range as the previous construct. ^b^ Fixed during the fitting procedure.

**Table 2:** Data collection and refinement statistics.

	H Type 1	H Type 3 (Globo H)		
**Data Collection**				
Beamline	FIP-BM30A (ESRF)	Proxima 1 (Soleil)		
Wavelength	0.98096	0.97857		
Space group	H32/R32 (H)	C2		
Unit cell dimensions (Å,°)	a = b = 42.7, c = 308.6	a = 74.4, b = 42.9, c = 102.6, β = 96.0		
Resolution (Å)	36.71–1.61 (1.64–1.61)	37.10–1.90 (1.94–1.90)		
Nb/nb unique reflections	113,880/14,606	87,318/25,081		
R_merge_	0.049 (0.588)	0.077 (0.363)		
R*_meas_*	0.056 (0.643)	0.105 (0.486)		
Mean I/σI	23.9 (3.7)	8.7 (2.9)		
Completeness (%)	99.7 (97.5)	97.9 (97.0)		
Redundancy	7.8 (7.7)	3.5 (3.5)		
CC 1/2	0.999 (0.874)	0.995 (0.861)		
**Refinement**				
Resolution (Å)	36.71–1.61	37.10–1.90		
Nb/nb free. reflections	14,605/751	25,080/1552		
*R*_work_/*R*_free_	15.8/20.3	16.5/22.7		
Rmsd Bond lengths (Å)	0.014	0.015		
Rmsd Bond angles (°)	1.78	2.0		
Rmsd Chiral (Å^3^)	0.099	0.102		
No. atoms/Bfac (Å^2^)	Chain A	Chain A	Chain B	Chain C
Protein	993/19.6	994/26.0	1027/25.9	1002/26.2
Ligand	36/23.2	47/33.6	58/41.6	58/38.8
Waters	135/28.3	109/32.2	106/31.7	82/32.5
Ramachandran Allowed (%)	100	100		
Favored (%)	97.8	96.9		
Outliers (%)	0	0		
PDB Code	6TID	6TIG		

Values in parentheses are for highest-resolution shell.

**Table 3:** Summary of the interactions of BC2L-CN2 with oligosaccharides.

Ligand Atom	Protein Atom or Water	Distance (Å) H-Type 1	Distance (Å) H-Type 3
**Fuc1**			
O2	Arg111 * NH2	3.06	3.04 ± 0.07
	Arg111 * NH1	2.88	3.03 ± 0.08
	HOH1 → Tyr 58 * OH	2.72 → 2.80	2.62 ± 0.08 → 3.01 ± 0.01
O3	Arg111 * NH2 *	3.18	3.15 ± 0.04
	Thr74 * OG1	2.54	2.62 ± 0.06
	HOH2 → Ser82 OG	2.59 → 2.66	2.60 ± 0.05 → 2.43 ± 0.03
	HOH2 → Tyr75 * O	2.59 → 2.64	2.60 ± 0.05 → 2.78 ± 0.01
O4	Arg85 NE	2.89	2.93 ± 0.02
	Thr83 O	2.69	2.71 ± 0.02
O5	Arg85 NH2	2.97	3.03 ± 0.08
C6	Tyr48	hydrophobic	hydrophobic
**GlcNAc3**			
O7	Ser82 OG	2.57	2.78 ± 0.07
	HOH3 → Thr83 N	3.01 → 3.02	3.00 ± 0.33→ 3.03 ± 0.13 (not in chain C)
N-acetyl	Tyr54 *	hydrophobic	hydrophobic
**Gal4**			
O4	HOH4 → Glu81 O		3.17 → 2.67 (chain C)
O6	HOH4 → Glu81 O		2.90 → 2.67 (chain C)
C1-C2	Phe54 *	hydrophobic	hydrophobic
**Gal5**			
O6	Lys78 * NZ		2.90 (chain C)

Residues from the neighboring protomer in the binding interface are labelled with an asterisk (*). For water-mediated interaction, an arrow is used to indicate which water is linked to which protein atom. For Globo H complex, mean distance and standard deviations were calculated from the distance in each protomer. Only distances less than 3.2 Å are listed.

**Table 4:** Dihedral angle measured for the observed glycosidic linkages of H-type 1 and Globo H.

	H-Type 1	Globo H		
Chain		A	B	C
	Fucα1-2Gal	Fucα1-2Gal		
Φ *	−78.9	−76.9	−78.8	−80.9
Ψ *	−101.5	−99.3	−100.5	−98.5
	Galβ1-3GlcNAc	Galβ1-3GalNAc		
Φ *	−73.3	−79.1	−66.9	−76.7
Ψ *	124.3	120.1	117.7	113.5
		GalNAcβ1-3Gal		
Φ *	NA	−78.8	−104.7	−92.6
Ψ *	NA	101.3	155.7	147.1
		Galα1-4Gal		
Φ *	NA	NA	72.2	79.1
Ψ *	NA	NA	98.1	100.5

* Φ (O5-C1-Ox-Cx) and Ψ (C1-Ox-Cx-Cx+1), in degrees (°) with x, the number of the carbon atoms of the second monosaccharide with which the 1 → x glycosidic bond was formed. NA: not applicable.
